# Sequential treatment of metastatic renal cell carcinoma patients after first-line vascular endothelial growth factor targeted therapy in a real-world setting: epidemiologic, noninterventional, retrospective–prospective cohort multicentre study

**DOI:** 10.1007/s00432-023-04645-x

**Published:** 2023-02-27

**Authors:** Alvydas Cesas, Vincas Urbonas, Skaiste Tulyte, Rasa Janciauskiene, Sigita Liutkauskiene, Ingrida Grabauskyte, Ignas Gaidamavicius

**Affiliations:** 1 Klaipeda University Hospital, Klaipeda, Lithuania; 2 National Cancer Institute, Vilnius, Lithuania; 3 Clinic of Internal Diseases, Family Medicine and Oncology, Faculty of Medicine, Institute of Clinical Medicine, Vilnius University, Vilnius, Lithuania; 4 Clinic of Oncology and Hematology, Institute of Oncology, Lithuanian University of Health Sciences Hospital of Lithuania, Kaunas Clinics Hospital, Kaunas, Lithuania; 5 Hospital of Oncology, Affiliate Hospital of Lithuanian University of Health Sciences, Kaunas, Lithuania; 6 Department of Physics, Mathematics and Biophysics, Lithuanian University of Health Sciences, Kaunas, Lithuania; 7 UAB “Guruma”, Kaunas, Lithuania

**Keywords:** mRCC, VEGF, PFS, PFS2, Treatment sequence, Real-world data

## Abstract

**Purpose:**

The purpose of our study was to determine whether data on the clinical effectiveness of second-line therapy collected in a real-world setting provide additional valuable information on the optimal sequence of metastatic renal cell carcinoma (mRCC) treatment.

**Methods:**

Patients diagnosed with mRCC who were treated with at least one dose of first-line vascular endothelial growth factor (VEGF)-targeted therapy with either sunitinib or pazopanib and with at least one dose of second-line everolimus, axitinib, nivolumab, or cabozantinib were included. The efficacy of different treatment sequences was analyzed based on the time to the second objective disease progression (PFS2) and the time to the first objective disease progression (PFS).

**Results:**

Data from 172 subjects were available for analysis. PFS2 was 23.29 months. The 1-year PFS2 rate was 85.3%, and the 3-year PFS2 rate was 25.9%. The 1-year overall survival rate was 97.0%, and the 3-year overall survival rate was 78.6%. Patients with a lower IMDC prognostic risk group had a significantly (*p* < 0.001) longer PFS2. Patients with metastases in the liver had a shorter PFS2 than patients with metastases in the other sites (*p* = 0.024). Patients with metastases in the lungs and lymph nodes (*p* = 0.045) and patients with metastases in the liver and bones (*p* = 0.030) had lower PFS2 rates than patients with metastases in other sites.

**Conclusions:**

Patients with a better IMDC prognosis have a longer PFS2. Metastases in the liver lead to a shorter PFS2 than metastases in other sites. One metastasis site means a longer PFS2 than 3 or more metastasis sites. Nephrectomy performed in an earlier stage of disease or metastatic setting means higher PFS and higher PFS2. No PFS2 difference was found between different treatment sequences of TKI–TKI or TKI-immune therapy.

## Background

Kidney cancer accounts for 3% and 5% of all adult malignancies in women and men, respectively, thus representing the 10th most common cancer in women and the 7th most common cancer in men (Escudier et al. 2019). According to the most recent version of the GLOBOCAN database, there were more than 431,288 cases of kidney cancer per annum diagnosed worldwide in 2020 (Sung et al. 2020). In Lithuania, 814 new cases of kidney cancer were diagnosed in 2020. Lithuania is among the countries with the highest age-standardized kidney cancer incidence rate, at 14.5 cases per 100,000 people, and the fifth highest overall mortality rate, at 4.2 cases per 100,000 people (Sung et al. 2020). Kidney cancer is a multifactorial disease, although the Chernobyl accident may be one of the reasons for the high renal cancer incidence (Marino and Nunziata 2018).

Three vascular endothelial growth factor (VEGF)-targeted agents have demonstrated first-line systemic treatment efficacy in pivotal phase III trials, mostly focused on favorable and intermediate-risk patients. At the beginning of our study, PI3-kinase/AKT/mTOR pathway inhibitors (everolimus), tyrosine kinase inhibitors (TKIs) (axitinib), PD-1 immune checkpoint inhibitors (nivolumab), and active novel multikinase inhibitors (cabozantinib) seemed to be attractive second-line treatment approaches after the use of first-line VEGF-targeted therapies in patients with advanced renal clear cell carcinoma (Escudier et al. 2019).

At the time of the real-world evidence data collection in Lithuania, the first-line systemic treatment of advanced renal cell carcinoma (RCC) included the VEGF-targeted agents sunitinib or pazopanib. Axitinib was the treatment option after the failure of sunitinib. According to national guidelines on RCC treatment, since August 2019, cabozantinib, everolimus or nivolumab have been deemed the second-line treatment options for advanced renal clear cell carcinoma after disease progression with first-line sunitinib or pazopanib therapy.

While the VEGF-receptor TKIs sunitinib and pazopanib have shown significant antitumour activity in locally invasive and metastatic renal cell carcinoma (mRCC), the times to disease progression of these agents were 8.4 months in the pazopanib group and 9.5 months in the sunitinib group (HR 1.05) (Motzer et al. 2013). Although the study showed noninferiority of progression-free survival (PFS), pazopanib was significantly favored in 11 different health-related quality of life measures. Patients treated with pazopanib had significantly less fatigue and foot soreness (Motzer et al. 2013). With the introduction of PD-1 immune checkpoint inhibitors (nivolumab) and active novel multikinase inhibitors (cabozantinib), the treatment options for mRCC have been significantly altered. However, the patient characteristics and survival outcomes in real-life clinical practice are dependent on access to treatment.

The data on comparative outcomes on the subsequent survival of mRCC patients are limited. More data in a real-life setting outside clinical trials are needed with regards to treatment pathways, sequencing, and the second objective disease progression-free survival after first-line VEGF-targeted therapy (e.g., sunitinib or pazopanib) in patients with locally invasive or mRCC receiving second-line treatment with everolimus, axitinib, nivolumab or cabozantinib.

There are several alternative schemes in the sequential treatment algorithm of mRCC. The time to the second objective disease progression (PFS2) is emerging as an endpoint in a growing number of clinical oncology trials assessing the benefits of maintenance or sequential treatment. PFS2 was defined as “time from randomization to objective tumor progression on second-line treatment or death from any cause”. In some cases, the time on next-line therapy may be used as a proxy for PFS. The European Medicines Agency (EMA) recommends using PFS2 to help understand the relevance of meaningful improvements in PFS when overall survival (OS) cannot be measured.

The objective of our study was to determine whether the PFS2 data collected in a real-world setting provide additional valuable information on the optimal mRCC treatment.

## Methods

The study aimed to cover national data. Patients were recruited from 5 centers throughout Lithuania. In this study, we gathered real-world data, which is why the sample size varies in the results.

Patients diagnosed with mRCC who were treated with at least one dose of first-line VEGF-targeted therapy with either sunitinib or pazopanib and with at least one dose of second-line everolimus, axitinib, nivolumab, or cabozantinib from January 1, 2017, to June 30, 2021, were included. Sunitinib and pazopanib were the only available agents in Lithuania for favorable- and intermediate-risk mRCC patients in 2017.

The collected data included patient demographic characteristics (sex, age, race, performance status, and risk factors), histological type, tumor characteristics, sites of metastasis, radiological examination results, type of received treatment, therapy efficacy, and sequences of the respective targeted agents. The patients were treated following the usual medical practice during their participation in this epidemiologic study.

The patients were stratified according to the international mRCC database consortium (IMDC, Heng) prognostic risk groups and the Memorial Sloan–Kettering Cancer Center (MSKCC, Motzer) score (at the time of this study, MSKCC was the main prognostic model). Data analysis was based on the IMDC prognostic risk groups.

The effectiveness of different treatment sequences was analyzed based on PFS2 and PFS. Safety was not evaluated in our study.

All statistical evaluations were performed using IBM SPSS Statistics 27. The primary efficacy variable PFS2, as well as the secondary efficacy variable PFS, are displayed by Kaplan–Meier curves. To compare quantitative variables between independent samples, Mann–Whitney *U* or Kruskal–Wallis tests (for non-normally distributed variables) were applied. To calculate correlations between two quantitative variables, Spearman correlation coefficients were applied.

## Results

### Patient data

**Fig. 1:**
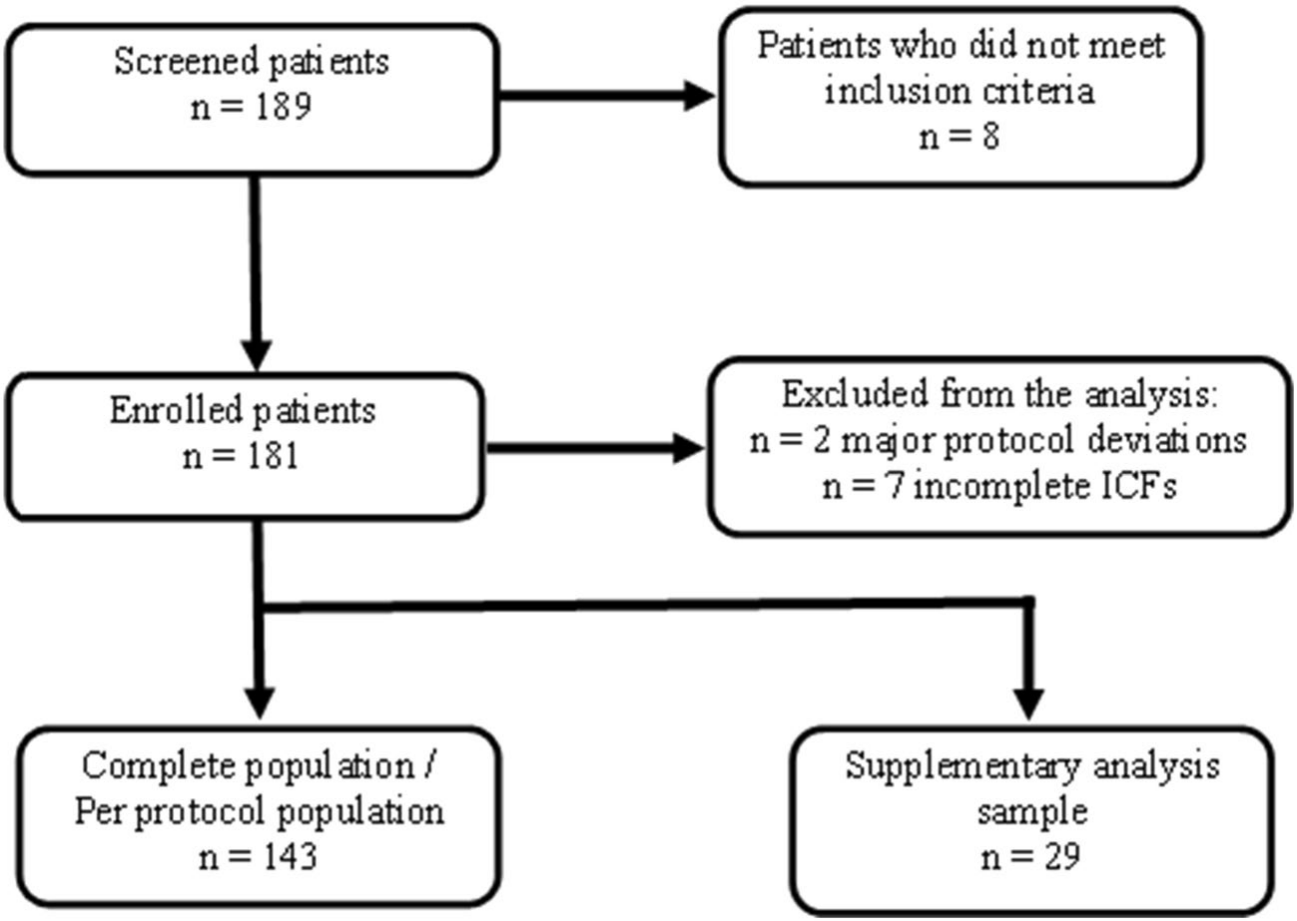
Study population analysis scheme

Data from 172 subjects were available for analysis. A total of 143 subjects completed the study, and their data were used for the main analysis. Twenty-nine subjects did not finish the second-line therapy at the end of follow-up, and their data were used for the supplementary analysis (Fig. 1).

### Patient characteristics

A total of 76.2% of patients were male. The median age at diagnosis was 67 years. All subjects were white/Caucasian (Table 1). Most of the patients had G2 (30.1%) and G3 (36.4) pathological grades by Fuhrman grading. The majority (55.2%) of patients had stage IV disease at the time of diagnosis.

**Table 1:** Demographic summary

	*N* = 143
Median age, years	67.0
Gender, *n* (%)	
Female	34 (23.8)
Male	109 (76.2)
Race, *n* (%)	
White/Caucasian	143 (100.0)
Black	0 (0.0)

The most common TNM stages in the sample were T3a (39.2%), N0 (44.8%), and M0 (52.4%). Metastases were detected in 52.4% of the patients at the time of diagnosis (Table 2). As prior treatment, 99 (69.2%) patients had undergone nephrectomy, and 50 (35.0%) patients had undergone palliative bone metastasis radiotherapy.

**Table 2:** Baseline disease characteristics

Pathological (Fuhrman) grade, *n* (%)	*N* = 139
Gx	35 (24.5)
G1	6 (4.2)
G2	43 (30.1)
G3	52 (36.4)
G4	3 (2.1)

Carcinoma stage, *n* (%)	*N* = 141
I	20 (14.0)
II	7 (4.9)
III	35 (24.5)
IV	79 (55.2)

Clinical stage (TNM), *n* (%)	*N* = 142
Tx	5 (3.5)
T1	10 (7.0)
T2	6 (4.2)
T3	21 (14.7)
T4	14 (9.8)
T1a	4 (2.8)
T2a	5 (3.5)
T3a	56 (39.2)
T1b	15 (10.5)
T2b	1 (0.7)
T3b	4 (2.8)
T3c	1 (0.7)
Nx	47 (32.9)
N0	64 (44.8)
N1	30 (21.0)
N2	1 (0.7)
Mx	9 (6.3)
M0	58 (40.6)
M1	75 (52.4)

Arterial hypertension was the most common comorbidity (18.9%). A total of 8.4% of patients were obese, and 1 (0.7%) patient had advanced kidney disease and analgesic abuse in anamnesis. None of the subjects had genetic or hereditary risk factors (Table 3).

**Table 3:** Demographic summary of medical risk factors

	*N* = 143
Obesity (BMI* ≥ 30), *n* (%)	
Obese	12 (8.4)
Nonobese	102 (71.3)
Unknown	29 (20.3)
Arterial hypertension (> 160/100 mmHg), *n* (%)	
Yes	27 (18.9)
No	87 (60.8)
Unknown	29 (20.3)
Family history of kidney disease, *n* (%)	
Absent	92 (64.3)
Unknown	51 (35.7)
Occupational exposure to toxic substances, *n* (%)	
Yes	3 (2.1)
No	83 (58.0)
Unknown	57 (39.9)
Analgesic abuse, *n* (%)	
Yes	1 (0.7)
No	87 (60.8)
Unknown	55 (38.5)
Advanced kidney disease, *n* (%)	
Yes	1 (0.7)
No	107 (74.8)
Unknown	35 (24.5)
Genetic and hereditary risk factors, *n* (%)	
No	85 (59.4)
Unknown	58 (40.6)

*BMI body mass index

Most of the patients were classified into the intermediate (60.8%) IMDC prognostic risk group and had MSKCC scores of 1 and 2 (30.1% and 32.9%) (Table 4).

**Table 4:** Demographics of MSKCC score and IMDC prognostic risk groups

Risk model	*N* = 143
IMDC, *n* (%)	
Favorable	32 (22.4)
Intermediate	87 (60.8)
Poor	16 (11.2)
Unknown	8 (5.6)
MSKCC, *n* (%)	
0	32 (22.4)
1	43 (30.1)
2	47 (32.9)
3	10 (7.0)
4	2 (1.4)
5	1 (0.7)
Unknown	8 (5.6)

### Treatment sequences

All enrolled patients received vascular endothelial growth factor-tyrosine kinase inhibitors (VEGF-TKIs) as first-line therapy. A total of 123 (86.1%) patients received sunitinib, and 20 (13.9%) received pazopanib as first-line therapy. Four different agents were used for second-line therapy: axitinib, nivolumab, cabozantinib, and everolimus. The most commonly used treatment sequences were sunitinib–axitinib (40.6%), sunitinib–nivolumab (26.6%), and sunitinib–cabozantinib (17.5%) (Table 5).

**Table 5:** Treatment sequences

Treatment sequence	*n* (%)
Sunitinib–cabozantinib	25 (17.5)
Sunitinib–nivolumab	38 (26.6)
Sunitinib–axitinib	58 (40.6)
Sunitinib–everolimus	2 (14)
Pazopanib–cabozantinib	5 (35)
Pazopanib–nivolumab	8 (5.6)
Pazopanib–axitinib	1 (0.7)
Pazopanib–everolimus	6 (4.2)

### Efficacy

**Fig. 2:**
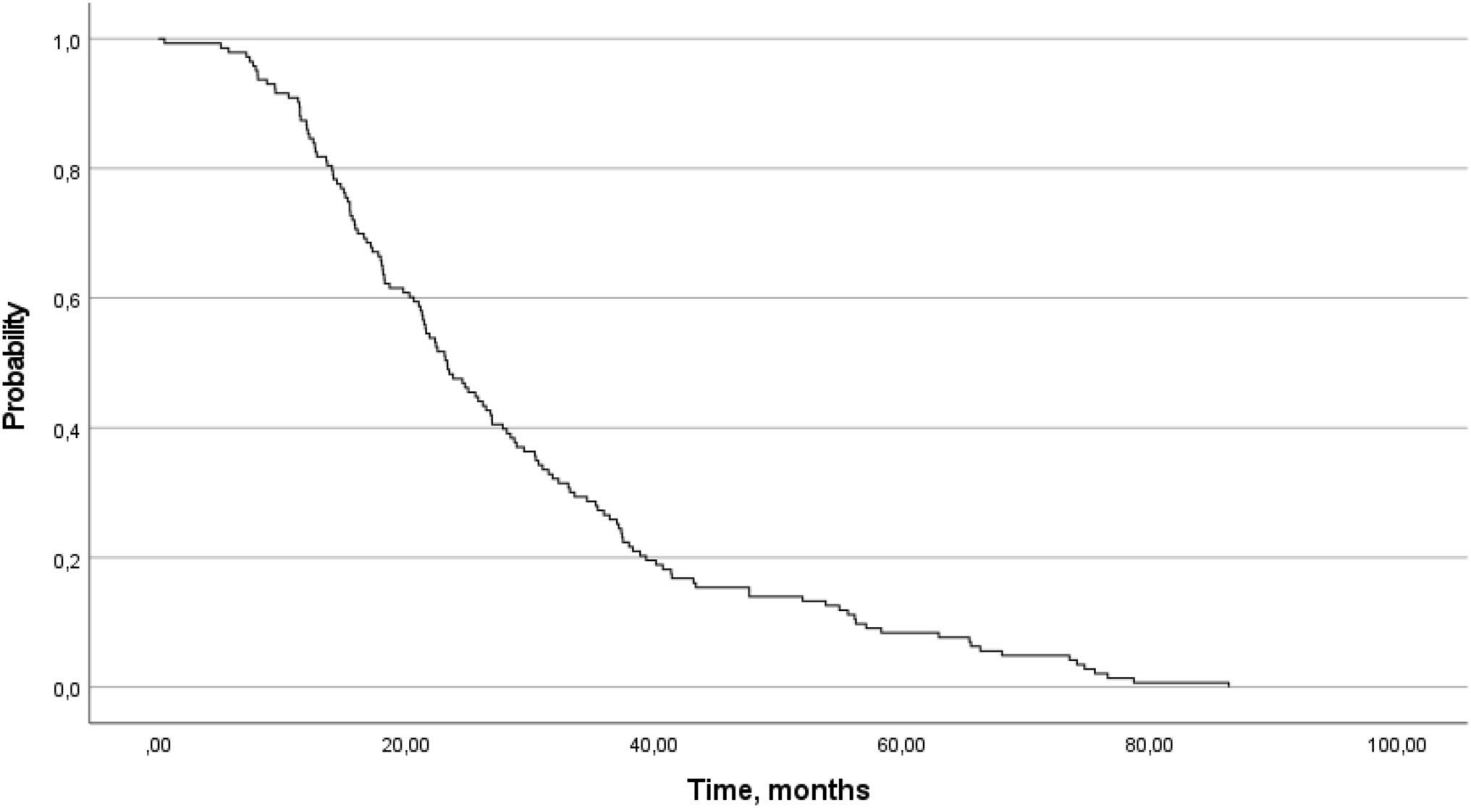
Progression-free survival on second-line treatment (Kaplan–Meier)

**Fig. 3:**
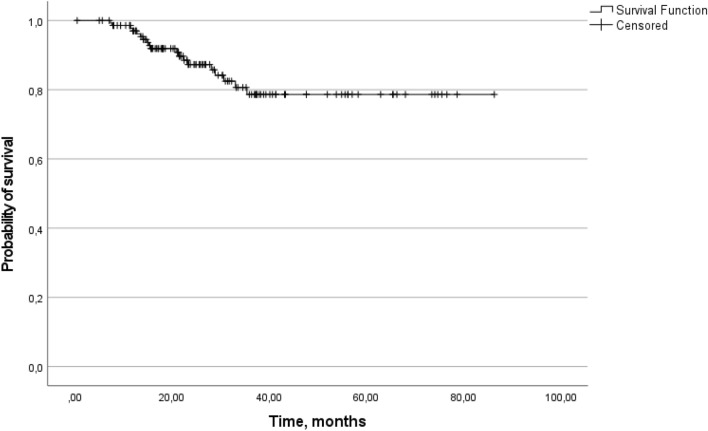
Overall survival (Kaplan–Meier)

The median time from day one of the first-line VEGF-targeted therapy to objective tumor progression on second-line treatment or death from any cause while on second-line treatment (PFS2) was 23.29 months. The 1-year PFS rate was 85.3%, and the 3-year PFS rate was 25.9% (Fig. 2). The 1-year OS rate was 97.0%, and the 3-year OS rate was 78.6%. The median OS was not reached, with a median follow-up time of 39.26 months (Fig. 3).

**Fig. 4:**
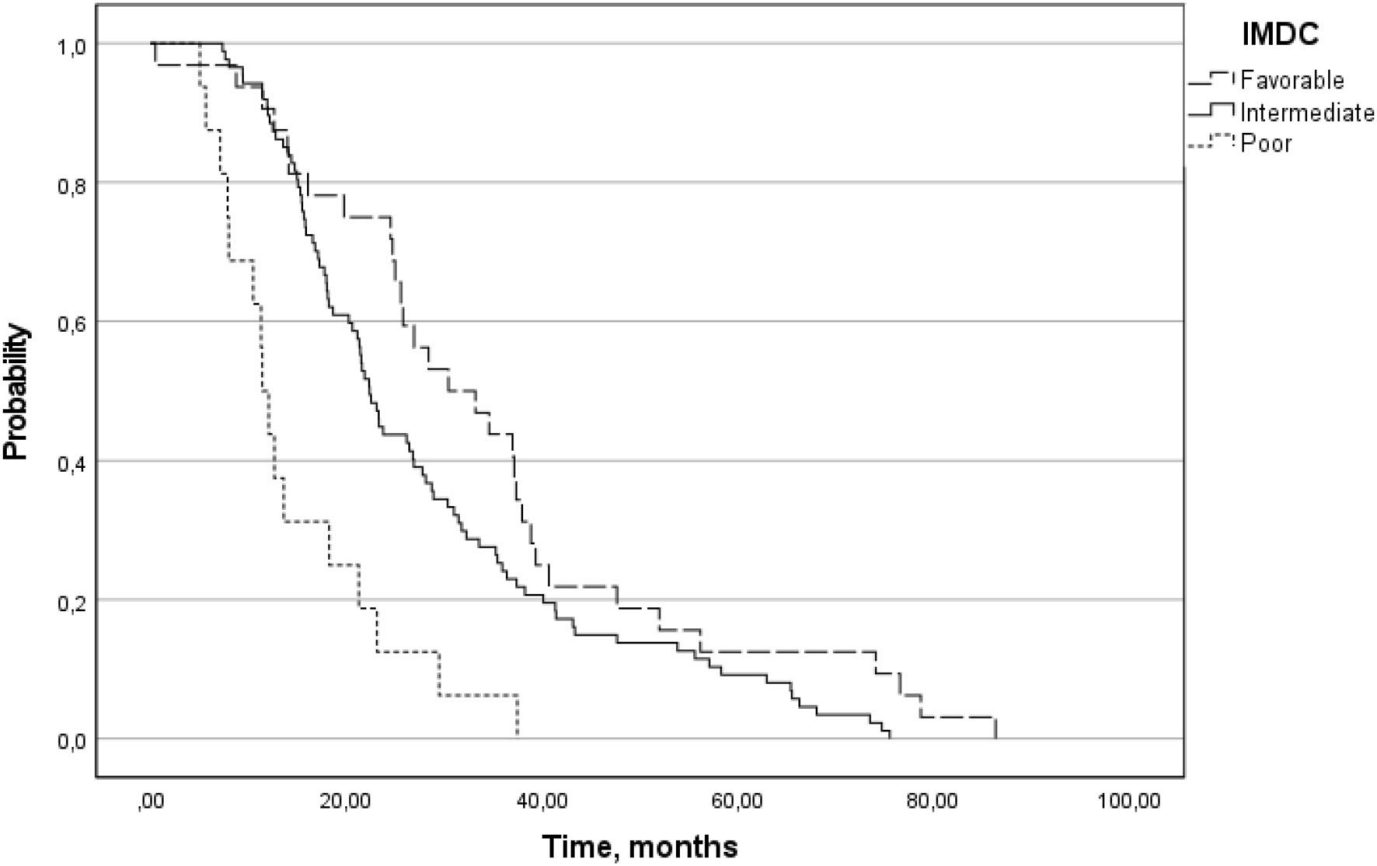
Comparison of the time to the second objective disease progression (PFS2) under second-line treatment, stratifying the patients by IMDC prognostic risk groups (Kaplan–Meier)

We evaluated and compared PFS2 based on the IMDC prognostic risk groups and therapy sequences. Patients in the favorable IMDC prognostic risk group had a significantly (*p* < 0.001) higher PFS2 (Table 6 and Fig. 4).

**Table 6:** Comparison of the time to the second objective disease progression (PFS2) under second-line treatment, stratifying the patients by IMDC prognostic risk groups

PFS2	Favorable	Intermediate	Poor
*n*	32	87	16
Median, months	31.80	22.37	11.72
Minimum, months	0.49	7.36	5.03
Maximum, months	86.31	75.50	37.45

We compared the PFS2 according to the first- and second-line treatment sequence, stratifying the patients by the IMDC prognostic risk groups and MSKCC score. The sunitinib–everolimus, pazopanib–axitinib, and pazopanib–everolimus treatment sequences were not included in the analysis because of the small sample size. No statistically significant PFS2 difference was found between different treatment sequences (all *p* > 0.05) (Table 7).

**Table 7:** Comparison of PFS2 stratified by treatment sequence and IMDC prognostic risk group

	Favorable	Intermediate	Poor
Sunitinib–cabozantinib			
*n* (%)	4 (12.5)	20 (23.0)	1 (6.3)
Median PFS2	27.15	21.55	NA
Sunitinib–nivolumab			
*n* (%)	5 (15.6)	25 (28.7)	7 (43.8)
Median PFS2	24.50	27.76	11.26
Sunitinib–axitinib			
*n* (%)	17 (53.1)	30 (34.5)	5 (31.3)
Median PFS2	33.18	23.19	12.05
Pazopanib–cabozantinib			
*n* (%)	1 (3.1)	3 (3.4)	1 (6.3)
Median PFS2	NA	18.00	NA
Pazopanib–nivolumab			
*n* (%)	3 (9.4)	4 (4.6)	1 (6.7)
Median PFS2	37.12	16.82	NA
*p* value	0.786	0.606	0.742

The median PFS was 15.15 months. We also found that surgical treatment resulted in a higher PFS and higher PFS2 (Table 8).

**Table 8:** Comparison of PFS and PFS2 based on surgical treatment

Nephrectomy	*n*	Median, months	*p* value
PFS			
No	41	11.10	**0.038**
Yes	99	17.05	
PFS2			
No	41	18.10	**0.015**
Yes	99	25.79	

### Supplementary analysis of radiological examination

Patients with no metastases in the lymph nodes (all diagnoses were supported histologically after nephrectomy) had a significantly (*p* = 0.008) longer PFS2 than patients with detected metastases in the lymph nodes.

Patients with metastases in the liver had a shorter PFS2 than patients with metastases in the other sites (*p* = 0.024). Patients with metastases in the lungs and lymph nodes (2 sites) (*p* = 0.045) and patients with metastases in the liver and bones (2 sites) (*p* = 0.030) had significantly lower PFS2 rates than patients with metastases in other sites.

Patients with 1 site of metastasis had a longer PFS2 than patients with 3 or more sites of metastasis (*p* = 0.034). PFS has a tendency (not statistically significant) to be longer in patients with 1 site of metastasis than in those with 3 or more sites of metastasis. We checked if there is a more effective treatment sequence for patients with different numbers of metastasis sites; however, no statistically significant differences were found.

## Discussion

This study assessed real-world treatment outcomes in patients with mRCC.

Many new agents have been introduced for mRCC treatment during the last decade. Newly available drugs enable many different treatment sequences. Nevertheless, it burdens the decision when choosing the treatment sequence in daily clinical practice as effective sequencing becomes crucial.

We compared PFS and PFS2 between different treatment sequences. None of the treatment sequences was significantly better than the others. No significant differences in PFS and PFS2 were observed between the different treatment sequences. In this case, the patient’s preference becomes more important, as they can choose the therapy considering the administration form, adverse reaction profile, availability, and local treatment guidelines.

Our study evaluated the prognostic value of the number and sites of metastases. We found that patients with liver metastases and more metastatic sites had lower PFS2 rates. Vincenzo Di Nunno et al. also proved that metastases in the liver leads to shorter survival rates (di Nunno et al. 2020).

The median PFS in our real-world data was 15.15 months, i.e., higher than for both pazopanib and sunitinib in randomized phase III clinical trials. Pazopanib has shown superiority against placebo in terms of the median PFS—9.2 months vs. 4.2 months (Sternberg et al. 2010). Sunitinib has shown a median PFS that was 6 months longer than that of interferon alpha (Motzer et al. 2007). A real-world data study in the Netherlands showed a similar median PFS of 15.7 months with pazopanib monotherapy, sunitinib monotherapy, or nivolumab–ipilimumab combination (van Laar et al. 2022).

The second-line treatment in Lithuania was available only beginning in July 2019. The most commonly used treatment sequences in our study were sunitinib–axitinib (40.6%), sunitinib–nivolumab (26.6%), and sunitinib–cabozantinib (17.5%).

All patients were treated with VEGF-TKIs as first-line therapy. The most commonly used sequences were TKI–TKI (62.3%) and TKI–immunotherapy (32.2%). A total of 86.0% of patients received sunitinib, and 14.0% received pazopanib for first-line therapy. A similar ratio was reported in another article—86% received sunitinib, and 14% received other TKIs (Maroun et al. 2018).

The median PFS was 15.15 months, and the median PFS2 was 23.29 months. The METEOR study showed a PFS advantage of cabozantinib over everolimus in second-line therapy (7.4 months vs. 3.9 months) (Choueiri et al. 2016). The AXIS study compared axitinib and sorafenib and found that axitinib led to significantly higher PFS in second-line therapy (6.7 months vs. 4.7 months) (Rini et al. 2011). Experience from a single institution in Greece indicated that axitinib is effective beyond second-line treatment (Tsironis et al. 2020). A study in Italy showed a PFS of 7.14 months for axitinib after sunitinib (Facchini et al. 2019). Comparing nivolumab and everolimus in patients who had one or two previous therapies, PFS was similar (4.6 months vs. 4.4 months) (Motzer et al. 2015). A different study showed a PFS of 13 months (Maroun et al. 2018). A retrospective review of medical records from US community oncology practices showed a median PFS of 10.8 months after the initiation of second-line therapy (Jonasch et al. 2014). The PFS in our study was longer by more than 4 months. Better management of adverse effects may be one of the reasons. A deeper analysis is needed to determine the reasons.

A total of 69.2% of our patients underwent surgery, and 35% underwent palliative radiotherapy, compared to 83% of patients who underwent surgical treatment and 10% of patients who underwent radiotherapy in other studies (Maroun et al. 2018). Most of the radiotherapy used in Lithuania was palliative and targeted at bone metastases. It helps to control bone metastasis-caused symptoms, increases the World Health Organization (WHO) performance status, and allows the use of first- and second-line treatments for a longer time. After the CARMENA trial and Systematic Review of the Role of Cytoreductive Nephrectomy, performing cytoreductive nephrectomy when metastases are detected at the same time is not recommended (Bhindi et al. 2019; Mejean et al. 2018). However, patients with primary nonmetastatic renal cancer who have undergone nephrectomy have a higher survival rate, which was also revealed in our study results.

The BIONIKK phase II trial assessed the molecular characteristics of the tumor in the context of mRCC and found that molecular characteristics can help differentiate patients into groups who need I-O doublets, nivolumab monotherapy or TKI monotherapy (Epaillard et al. 2020). Our results confirm TKIs as an option for first-line treatment, with a maximum PFS2 in the favorable-risk group of 86.31 months. In addition, our study shows that clinical factors such as prior surgical treatment, IMDC risk group, number of metastasis sites, and metastasis localization are important when choosing therapy.

The variety of treatment combinations has made treatment decisions challenging. Front-line therapy decisions are still crucial when immunotherapy–TKIs are not available for all IMDC risk groups. On the other hand, the nivolumab–cabozantinib combination in CHECKMATE 9ER showed a median PFS of 16.6 months (Motzer et al. 2018), while the median PFS in our study was 15.15 months. The other limitations of combination therapy are a higher rate of adverse reactions and uncertainty of the next-line therapy after combination. Zarrabi et al. (2022) reported that the TKI–nivolumab combination may be a better choice than double immune checkpoint inhibition in favorable-risk patients.

The small sample for different treatment sequences was a limitation of our clinical study. A larger sample is needed to prove the tendencies observed in our study.

Currently, the treatment of patients in intermediate- and poor-risk groups remains indefinite. This group of patients would benefit the most from predictive molecular and genetic factors that could show sensitivity to angiogenesis inhibition, PD-1 inhibitors, or double immune blockade.

Many clinical factors, such as prognostic groups, sites of metastases, and the number of organs affected by metastases, are known to have prognostic value and could aid in the selection of optimal treatment patterns from available options. Despite the progress made in mRCC management, the disease eventually affects patients’ quality of life and survival. The high variability of RCC subtypes makes it a candidate for a personalized medicine approach (Sharma et al. 2022). Hence, discovering genetic and molecular biomarkers is crucial for precision therapy in mRCC.

Since Lithuania has one of the highest renal carcinoma incidence rates, a clinical trial such as this one was necessary to reveal the strategies of mRCC treatment in our country. The discovery that there is no PFS2 difference between second-line agents will be significant in future mRCC treatment. A small sample of subjects (all mRCC patients in Lithuania) and the fact that second-line treatment was available only since 2019 were the biggest limitations of our study.

## Conclusions

Patients with a better IMDC prognosis have a longer PFS2. Metastases in the liver lead to a shorter PFS2 than metastases in other sites. One site of metastasis means a longer PFS2 than 3 or more sites of metastasis. Nephrectomy performed in an earlier stage of disease or in metastatic setting means a higher PFS and higher PFS2. No PFS2 difference was found between different treatment sequences of TKI–TKI and TKI–immune therapy.

## Data Availability

The datasets generated and/or analyzed during the current study are available from the corresponding author on reasonable request.
